# Risk factors of acute bacterial paranasal sinusitis in children: a case control study

**DOI:** 10.1186/s12879-025-11299-2

**Published:** 2025-08-22

**Authors:** Ting-Fang Chiu, Ya-Li Hu, Jung-Chieh Du, Tsung-Zu Wu, Jason Jiunshiou Lee, Ping-Ing Lee

**Affiliations:** 1 Department of Pediatrics, Taipei City Hospital, Zhongxiao Branch, Taipei, Taiwan; 2 Department of Health and Welfare, University of Taipei, Taipei, Taiwan; 3 School of Medicine, National Yang Ming Chiao Tung University, Taipei, Taiwan; 4 Graduate Institute of Business Administration, Fu Jen Catholic University, New Taipei, Taiwan; 5 Department of Pediatrics, Cathay General Hospital, Taipei, Taiwan; 6 Department of Pediatrics, National Taiwan University Hospital and National Taiwan University College of Medicine, Taipei, Taiwan; 7 Department of Pediatrics, Taipei City Hospital, Heping-Fuyou Branch (Fuyou), Taipei, Taiwan; 8 Department of Family Medicine, Taipei City Hospital, Yangming Branch, Taipei, Taiwan; 9 Department of Pediatrics, National Taiwan University Children’s Hospital, No.8, Chung Shan S. Rd., Taipei, 10041 Taiwan

**Keywords:** Acute paranasal sinusitis, Allergic rhinitis, Nose blowing, Nasal irrigation, Incense

## Abstract

**Background:**

Acute bacterial paranasal sinusitis is a common infection in children. The aim of this study was to analyze risk factors associated with acute bacterial paranasal sinusitis in children.

**Methods:**

Children aged 4 to 18 years were enrolled and received questionnaire survey from January 2020 to December 2021. Participants with a diagnosis of acute bacterial paranasal sinusitis received antibiotic treatment and were followed to evaluate the treatment outcome.

**Results:**

A total of 228 participants was enrolled and divided to 3 groups, including acute bacterial paranasal sinusitis, allergic rhinitis without acute bacterial paranasal sinusitis and control group. Children having acute bacterial paranasal sinusitis were significantly younger than those who did not have (*p* = 0.02). Children with acute bacterial paranasal sinusitis had significantly higher rates of residential incense burning every day (*p* = 0.01) and antibiotic use within three months (*p* < 0.001). Multivariable logistic regression analysis showed that incense burning every day (aOR 2.45 [95% CI 1.25, 4.80], *p* < 0.001) and antibiotic use within three months (aOR 8.04 [95% CI 3.92, 16.50], *p* < 0.001) were significant risk factors of acute bacterial paranasal sinusitis. Nose blowing did not predispose to acute bacterial paranasal sinusitis, and did correlate with a favorable treatment response. Nasal irrigation was not beneficial to antibiotic treatment response of sinusitis.

**Conclusion:**

Exposure to residential incense and antibiotic use within three months were significant risk factors for acute bacterial paranasal sinusitis in children. Nose blowing exhibited no detrimental effect, while nasal irrigation showed no significant beneficial effect on either the occurrence or treatment outcome.

**Supplementary Information:**

The online version contains supplementary material available at 10.1186/s12879-025-11299-2.

## Introduction

Acute bacterial paranasal sinusitis (ABPS) is a common complication of upper respiratory tract infection and allergic rhinitis. The prevalence rate is estimated to be 5% to 10% in children [1–4]. The definition of ABPS remained controversial. According to the American Academy of Pediatrics clinical practice guideline [4], children having acute upper respiratory tract infection with (1) persistent illness lasting for more than ten days without improvement, (2) worsening course after initial improvement or (3) severe onset for at least three consecutive days could be diagnosed of acute bacterial sinusitis. The leading pathogens of acute bacterial paranasal sinusitis include *Streptococcus pneumoniae*, nontypeable *Haemophilus influenzae*, and *Moraxella catarrhalis* [4, 5].

Allergic rhinitis is an important predisposing factor of ABPS [3, 6]. It had been reported that patients with perennial allergic rhinitis had a higher prevalence of sinusitis than those with seasonal allergic rhinitis, especially in children above 6.5 years [7]. Previous studies also found that passive smoking in the household was a risk factor of ABPS among pediatric population [1, 8, 9]. The pneumococcal conjugate vaccine (PCV) has been found to be beneficial in the prevention of acute sinusitis in children. One study in Sweden showed that PCV led to a 66% lower risk of hospitalization for sinusitis in children younger than two years [10]. Another study indicated that the incidence of acute sinusitis decreased significantly in both pediatric outpatient and inpatient setting after the launch of 13-valent PCV program [11]. Nevertheless, the relationship between additional household factors (such as exposure to residential incense and having pets at home), personal behaviors for relieving nasal symptoms (including nose blowing and nasal irrigation), and ABPS remained uncertain.

A study suggested that bacteria colonized in the nasopharynx may be propelled into the maxillary sinus after nose blowing [12]. Avoiding nose blowing could thus be considered a reasonable measure to prevent ABPS. Nasal irrigation is recommended for relieving symptoms in children with acute upper respiratory tract infections and acute sinusitis [13–16]. However, there is a lack of clinical data to either support or refute these recommendations.

Acute paranasal sinusitis could progress to chronic rhinosinusitis which was defined as symptoms lasting for more than 90 days [3]. Treating ABPS properly is essential to prevent recurrent or chronic sinusitis [17]. Understanding and avoiding predisposing factors of acute bacterial sinusitis may benefit the health and the life quality. The aim of this study was to analyze the risk factors for acute bacterial paranasal sinusitis in children.

## Materials and methods

We enrolled children aged 4 to 18 years old who visited the pediatric outpatient department of Zhongxiao and Heping-Fuyou branch of Taipei City Hospital from January 2020 to December 2021. All participants received physician consultation and questionnaire (supplementary 1) investigation after they and/or their guardians signed the patient informed consent. This study was approved by Institutional Review Board of Taipei City Hospital (No.TCHIRB-10901005-E). Questionnaire analysis collected the information of allergic history, daily behavior for managing nasal symptoms, medication use, home environment including pet, smoke or incense exposure, number of household members and vaccination record. The frequency of nasal irrigation and nose blowing was classified into never, seldom (less than once per day in average), often (once per day) and very frequent (more than twice per day).

The participants were divided into ABPS (group1), allergic rhinitis without ABPS (group 2) and control group (no allergic rhinitis and no ABPS). Exclusion criteria included immunodeficiency, malignancy, congenital abnormality of facial development, previous head surgery and ABPS lasting for more than 28 days. The definition of ABPS was based on the recommendations for the diagnosis and management of ABPS in children from the Pediatric Infectious Diseases Society of Taiwan [18]. Children presenting any of the following symptoms could be diagnosed with ABPS: (1) yellowish-greenish purulent nasal discharge persisted for more than 72 h, especially with co-existing fever, (2) yellowish-greenish purulent nasal discharge or severe stuffy nose, accompanied by new onset halitosis, (3) worsening of fever, headache, yellowish-greenish purulent nasal discharge after initial improvement, and (4) rhinorrhea or stuffy nose accompanied with tenderness and erythematous change at maxillary, frontal, or ethmoidal sinus area. The clinical manifestations and duration of fever, rhinorrhea, stuffy nose, headache, halitosis and cough were recorded. Outpatient department follow up was arranged every three days after initiation of antibiotic treatment to evaluate the treatment response. The first line antibiotic treatment was amoxicillin/clavulanate. Antibiotic treatment would be changed to cefixime, ceftibuten, trimethoprim-sulfamethxazole, ciprofloxacin or levofloxacin according to nasal discharge culture results and clinical response three days after the first line regimen. The antibiotic treatment course was 14 days and prolonged treatment for more than 14 days was defined as a poor treatment response.

### Statistical analysis

We employed chi-square tests or Fisher’s exact tests to analyze differences in categorical variables among groups. For continuous variables, we utilized the Kruskal–Wallis test, Mann–Whitney U test, or ANOVA test, depending on the distribution of the data. Multivariable logistic regression analysis was conducted to identify the risk factors associated with ABPS and poor antibiotic treatment response. Adjusted odds ratios with 95% confidence intervals (CI) for binary outcomes were calculated. Statistical analyses were performed using SAS version 9.4, and significance was set at *P* < 0.05.

## Results

A total of 267 participants was divided into group 1 with ABPS (*N* = 76), group 2 with allergic rhinitis and without ABPS (*N* = 109), and control group (*N* = 82). The basic data of all participants were summarized in Table 1. Children having ABPS were significantly younger than those who did not have (*p* = 0.02). In the control group, only one child had allergic conjunctivitis and none had a history of atopic disorders. Group 2 participants had significantly higher rates of allergic conjunctivitis. There was no significant difference in gender, parental education level and residence among these three groups.

**Table 1:** Basic information of three group of children

	Group 1 *N* = 76	Group 2 *N* = 109	Control group *N* = 82	*P* value
Sex				
Boy	37 (48.7%)	61 (56.0%)	38 (46.3%)	0.38
Girl	39 (51.3%)	48 (44.0%)	44 (53.7%)	
Age (years) (median, IQR)	6 (5–8.5)	7 (5–10)	7 (5–11)	0.02^*^
Atopic disease				
Atopic dermatitis	14 (18.4%)	30 (27.5%)	0 (0%)	< 0.001^*^
Allergic conjunctivitis	31 (40.8%)	83 (76.2%)	1 (1.2%)	< 0.001^*^
Allergic rhinitis	59 (77.6%)	109 (100%)	0 (0%)	< 0.001^*^
Asthma	24 (31.6%)	41 (37.6%)	0 (0%)	< 0.001^*^
Paternal education level				
Illiterate or elementary	0 (0%)	0 (0%)	0 (0%)	0.63
Junior high school	2 (2.6%)	4 (3.7%)	6 (7.3%)	
Senior high school	15 (19.7%)	17 (15.6%)	11 (13.4%)	
College	41 (53.9%)	59 (54.1%)	39 (47.6%)	
Graduate school	18 (23.7%)	29 (26.6%)	26 (31.7%)	
Maternal education level				
Illiterate or elementary	1 (1.3%)	0 (0%)	2 (2.4%)	0.47
Junior high school	2 (2.6%)	3 (2.8%)	3 (3.7%)	
Senior high school	15 (19.7%)	13 (11.9%)	11 (13.4%)	
College	46 (60.5%)	78 (71.6%)	49 (59.8%)	
Graduate school	12 (15.8%)	15 (13.8%)	17 (20.7%)	
Residence				
Taipei City	60 (78.9%)	95 (87.2%)	64 (78.1%)	0.17
New Taipei City	15 (19.7%)	12 (11.0%)	17 (20.7%)	
Hsinchu County	1 (1.3%)	2 (1.8%)	0 (0%)	
Taichung City	0 (0%)	0 (0%)	1 (1.2%)	

Group 1: acute bacterial paranasal sinusitis; group 2: allergic rhinitis without acute bacterial paranasal sinusitis; control group: no allergic rhinitis and acute bacterial paranasal sinusitis

^*^p < 0.05

Children diagnosed with ABPS exhibited significantly higher rates of daily residential incense burning (*p* = 0.01) and antibiotic use within three months (*p* < 0.01). Comparatively, the control group showed a higher frequency of nose blowing than both group 1 (*p* = 0.007) and group 2 (*p* = 0.01). However, there was no significant difference in the frequency of nose blowing between group 1 and group 2. Moreover, no significant differences were observed in the type of daycare, number of household members, exposure to smoke, or presence of pets at home among the three groups (Table 2).

**Table 2:** Correlation between acute bacterial paranasal sinusitis and personal behavior/lifestyle

	Group 1 *N* = 76	Group 2 *N* = 109	Control group *N* = 82	*P* value
Children blow the noses when having nasal symptoms				
Never or seldom	14 (18.4%)	18 (16.5%)	4 (4.9%)	0.02^*^
Often or very frequently	62 (81.6%)	91 (83.5%)	78 (95.1%)	
Children irrigate the noses when having nasal symptoms				
Never or seldom	69 (90.8%)	102 (93.6%)	81 (98.8%)	0.06
Often or very frequently	7 (9.2%)	7 (6.4%)	1 (1.2%)	
Type of day care				
Home care	2 (2.6%)	4 (3.7%)	2 (2.4%)	0.32
Kindergarten	41 (54.0%)	43 (39.5%)	33 (40.2%)	
School	33 (43.4%)	62 (56.9%)	47 (57.3%)	
Number of household members < 19 years (median, IQR)	2 (1–2)	2 (2)	2 (2)	0.21
Number of household members ≥ 19 years (median, IQR)	2 (2–4)	2 (2–3)	2 (2–3)	0.94
Residential incense burning				
Every day	27 (35.5%)	18 (16.5%)	21 (25.9%)	0.01^*^
Never or not every day	49 (64.5%)	91 (83.5%)	60 (74.1%)	
Residential smoke exposure				
Yes	15 (19.7%)	23 (21.1%)	22 (26.8%)	0.51
No	61 (80.3%)	86 (78.9%)	60 (73.2%)	
Pet at home				
No	63 (82.9%)	90 (82.6%)	74 (90.2%)	0.59
Dog	5 (6.6%)	11 (10.1%)	3 (3.7%)	
Cat	6 (7.9%)	5 (4.6%)	4 (4.9%)	
Other animal	2 (2.6%)	3 (2.8%)	1 (1.2%)	
Antibiotic use within three months				
Yes	33 (43.4%)	13 (11.9%)	4 (4.9%)	< 0.001^*^
No or unsure	43 (56.6%)	96 (88.1%)	78 (95.1%)	
13-valent PCV				
≥ 3 doses	68 (89.5%)	97 (89.0%)	71 (86.6%)	0.82
< 3 doses or unsure	8 (10.5%)	12 (11.0%)	11 (13.4%)	
Influenza vaccination				
Every year	44 (57.9%)	73 (67.0%)	45 (54.9%)	0.20
Irregular or never	32 (42.1%)	36 (33.0%)	37 (45.1%)	

Group 1: acute bacterial paranasal sinusitis; group 2: allergic rhinitis without acute bacterial paranasal sinusitis; control group: no allergic rhinitis and acute bacterial paranasal sinusitis

^*^p < 0.05

### Personal behavior and antibiotic treatment response in group 1

Among children diagnosed with ABPS, there was no significant difference in antibiotic use between those who never or seldom blew their nose (*n* = 14) and those who often or very frequently blew their nose (*n* = 62). In group 1 children, first-line antibiotic treatment failed in 11 out of 76 cases (14.5%; Table 3). Second-line antibiotic treatment was initiated for these cases due to symptoms such as purulent rhinorrhea, severe nasal congestion, or fever persisting for more than three days after initial therapy. Nasal irrigation did not correlate with the type of antibiotic treatment received. Those who often or very frequently irrigated their nose showed a relatively higher incidence of first-line antibiotic treatment failure and required a more prolonged duration of antibiotic treatment, although this difference was not statistically significant (Table 3).

**Table 3:** The association between personal behavior and antibiotic treatment response in children with acute bacterial paranasal sinusitis

	**Never or seldom**	**Often or very frequently**	***P* value**
A. Nose blowing	*N* = 14	*N* = 62	
First line antibiotic use	13 (92.9%)	53 (85.5%)	0.34
First line antibiotic treatment duration (days) (median, IQR)	14 (8–14)	10 (7–14)	0.08
Second line antibiotic use	3 (21.4%)	8 (12.9%)	0.35
Second line antibiotic treatment duration (days) (median, IQR)	14 (7–14)	11.5 (8.5–14)	1.0
Antibiotic use > 14 days	5 (35.7%)	7 (11.3%)	0.03
B. Nasal irrigation	*N* = 69	*N* = 7	
First line antibiotic use	61 (88.4%)	6 (85.7%)	0.56
First line antibiotic treatment duration (days) (median, IQR)	10 (7–14)	12.5 (12–14)	0.19
Second line antibiotic use	10 (14.5%)	1 (14.3%)	1.0
Second line antibiotic treatment duration (days) (median, IQR)	11.5 (8–14)	21.0 (21.0)	0.13
Antibiotic use > 14 days	10 (14.5%)	2 (28.6%)	0.25

First line antibiotic referred to amoxicillin/clavulanate. Second line antibiotics included cefixime, ceftibuten, ciprofloxacin, levofloxacin, and trimethoprim-sulfamethxazole. The regimen was prescribed based on clinical treatment response and nasal discharge culture result

Children with poor antibiotic treatment responses exhibited significantly higher rates of never or seldom blowing their noses compared to those with more favorable treatment responses (41.7% versus 13.3%, *p* = 0.03). Additionally, the median age of children with poor treatment responses (7 years, IQR 6–10) was significantly older than that of children with more favorable treatment responses (5 years, IQR 4–8, *p* = 0.03). Excluding children younger than five years, who might not be able to blow their noses properly, it was observed that group 1 children who never or seldom blew their noses tended to have a poor antibiotic treatment response (*p* = 0.03).

In the multivariable logistic regression analysis of group 1 data, never or seldom blowing the nose emerged as a significant risk factor for poor antibiotic treatment response in ABPS (aOR 7.26, 95% CI [1.4, 37.6], *p* = 0.018). Gender, age, frequency of nasal irrigation, atopic history, and exposure to smoke or incense did not have a significant impact on antibiotic treatment response.

### Risk factors of acute bacterial paranasal sinusitis

**Fig. 1:**
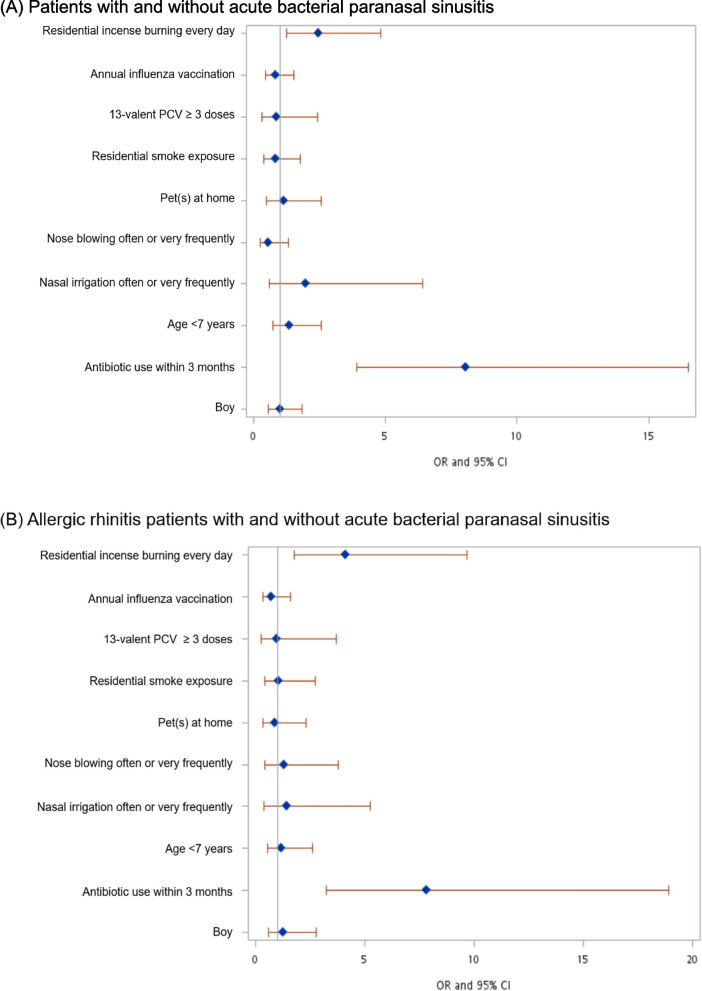
Multivariable logistic regression analysis of risk factors of acute bacterial paranasal sinusitis in all patients (**A**) and allergic rhinitis patients (**B**)

Participants with ABPS were significantly younger than those without (*p* < 0.01). Age younger than seven years (61.8% versus 48.2%, OR 1.74, 95% CI [1.01, 3.00], *p* = 0.04), daily residential incense burning (35.5% versus 20.5%, OR 2.13, 95% CI [1.19, 3.84], *p* = 0.01), and antibiotic use within three months (43.3% versus 8.9%, OR 7.86, 95% CI [4.00, 15.41], *p* < 0.01) were significant risk factors for ABPS (Table 4). Multivariable logistic regression analysis revealed that children in group 1 had a significantly higher risk of daily residential incense burning (aOR 2.45, 95% CI [1.25, 4.80], *p* < 0.01) and antibiotic use within three months (aOR 8.04, 95% CI [3.92, 16.50], *p* < 0.01) (Fig. 1). For children with allergic rhinitis, multivariable logistic regression analysis showed that daily residential incense burning (39% versus 16.5%, aOR 4.12, 95% CI [1.76, 7.66], *p* < 0.01) and antibiotic use within three months (45.8% versus 11.9%, aOR 7.84, 95% CI [3.25, 18.9], *p* < 0.01) were significant risk factors for ABPS (Fig. 1).

**Table 4:** Risk factors associated with acute bacterial paranasal sinusitis by univariable and multivariable logistic regression analysis

	Sinusitis (*N* = 76)	No sinusitis (*N* = 191)	OR (95% CI)	*P* value	aOR (95% CI)	*P* value
Age (median, IQR)	6 (5–8.5)	7 (5–11)		0.01^*^		
Age < 7y/o	47 (61.8%)	92 (48.2%)	1.74 (1.01, 3.00)	0.04^*^	1.36 (0.72, 2.56)	0.33
Sex						
Boy	37 (48.7%)	99 (51.8%)	1.13 (0.67, 1.93)	0.64	0.99 (0.54, 16.4)	0.99
Girl	39 (51.3%)	92 (48.2%)				
Number of household members						
< 19y/o (median, IQR)	2 (1–2)	2 (2)		0.08		
≧19y/o (median, IQR)	2 (2–4)	2 (2–3)		0.83		
Nasal irrigation						
Often or very frequently	7 (9.2%)	7 (3.7%)	0.38 (0.13, 1.11)	0.08	2.22 (0.66, 7.45)	0.19
Never or seldom	69 (90.8%)	184 (96.3%)				
Nose blowing						
Often or very frequently	62 (81.6%)	169 (88.5%)	0.58 (0.28, 1.19)	0.14	0.58 (0.25, 1.32)	0.19
Never or seldom	14 (18.4%)	22 (11.5%)				
Residential incense burning						
Every day	27 (35.5%)	39 (20.5%)	2.13 (1.19, 3.84)	0.01^*^	2.45 (1.25, 4.80)	< 0.001^*^
Never or not every day	49 (64.5%)	151 (79.5%)				
Residential smoke exposure						
Yes	15 (19.7%)	45 (23.6%)	0.79 (0.41, 1.54)	0.49	0.84 (0.39, 1.78)	0.65
No	61 (80.3%)	146 (76.4%)				
Pet at home						
Yes	13 (17.1%)	28 (14.7%)	1.20 (0.59, 2.47)	0.62	1.14 (0.51, 2.57)	0.75
No	63 (82.9%)	163 (85.3%)				
Antibiotic use within three months						
Yes	33 (43.3%)	17 (8.9%)	7.86 (4.00, 15.41)	< 0.001^*^	8.04 (3.92, 16.50)	< 0.001^*^
No or unsure	43 (56.6%)	174 (91.1%)				
13-valent PCV						
≥ 3 doses	68 (89.5%)	168 (88.0%)	1.16 (0.49, 2.73)	0.73	0.87 (0.31, 2.41)	0.79
< 3 doses or unsure	8 (10.5%)	23 (12.0%)				
Influenza vaccination						
Every year	44 (57.9%)	118 (61.8%)	0.85 (0.49, 1.46)	0.56	0.82 (0.44, 1.52)	0.53
Irregular or never	32 (42.1%)	73 (38.2%)

## Discussion

Most group 1 patients had allergic rhinitis, while this is not true for children in the control group. As can be expected, allergic rhinitis is a very important predisposing factor for ABPS. Residential incense burning every day and antibiotic use within three months were important risk factors of ABPS in children. Previous studies found that incense burning had adverse effects on pediatric lung function, and may predispose to bronchitis, bronchiolitis, pneumonia, wheezing and asthma [19–21]. Incense exposure is also related to many respiratory, cardiovascular, neuropsychological disease, and even cancer [22]. The complex mixture of chemicals and irritants in incense might cause nasal irritation, secretions or congestion [22, 23]. The present study emphasizes again the important role of incense burning in inducing ABPS in children.

Antibiotic treatment is typically recommended for 10 to 14 days for ABPS to expedite disease resolution, increase cure rates, and prevent complications [4, 24]. We found that antibiotic use within three months was a significant risk factor for ABPS. Recurrent acute sinusitis is not uncommon [25, 26], with several investigators suggesting that predisposing factors may include recurrent viral infections, abnormal glandular secretions, and underlying mucosal abnormalities [26, 27]. The association between previous antibiotic use and ABPS may be attributed to individual factors predisposing to bacterial mucosal infection, such as anatomical defects, immune-related susceptibility, and exposure to incense burning. Another possible explanation is that some cases of bacterial sinusitis in our study may represent recurrent infections following previous episodes due to inappropriate durations of prior antibiotic treatment or antibiotic-resistant bacteria.

Nasal irrigation is recommended for adults with chronic paranasal sinusitis to alleviate symptoms [13, 28, 29]. It is also considered safe and beneficial for children with acute upper respiratory tract infections and acute sinusitis, primarily providing symptomatic relief [13–16]. However, there is limited evidence supporting its impact on antibiotic effectiveness, treatment duration, or sinusitis recurrence. A systematic review by Cochrane found no significant evidence supporting the efficacy of nasal irrigation for acute sinusitis [30]. In our study, we observed that individuals who irrigated their nose often or very frequently tended to have a longer duration of antibiotic treatment, although this difference was not statistically significant. The impact of nasal irrigation on the treatment response of ABPS in children warrants further investigation.

Nose blowing, a common practice to clear nasal secretions, carries potential risks such as mucosal injury, epistaxis [31], pneumocephalus [32], pneumolabyrinth [33], and orbital blowout fractures [34]. A study utilizing sinus computerized tomography revealed that radiopaque contrast medium from the nasopharynx may be propelled into the maxillary sinus during nose blowing [12]. While avoiding nose blowing might seem like a preventive measure for ABPS, there is currently no clinical evidence to support this suggestion. Contrary to expectations, our study findings indicate that nose blowing is not associated with an increased risk of ABPS in children. In fact, nose blowing appears to be correlated with favorable antibiotic responses. However, further research is necessary to confirm these findings.

This study has several limitations. Firstly, the questionnaire survey may be susceptible to recall bias, as participants may not accurately recall past events or behaviors. Secondly, we were unable to measure the concentration and duration of residential incense exposure, which could potentially affect the results. Finally, the study lacked comprehensive medical information regarding previous antibiotic use, including the indication and diagnosis, which may have influenced the interpretation of results.

## Conclusion

In conclusion, residential incense exposure and antibiotic use within three months were significant risk factors of ABPS in children. Nose blowing exhibited no detrimental effect, while nasal irrigation showed no significant beneficial effect on either the occurrence or treatment outcome.

## Supplementary Information

Supplementary Material 1.

## Data Availability

Data from this study will be provided upon reasonable request to the corresponding author.

## Abbreviations

ABPS: Acute bacterial paranasal sinusitis
PCV: Pneumococcal conjugate vaccine
