# Supplementary material for: Risk factors of acute bacterial paranasal sinusitis in children: a case control study
Source: BMC Infect Dis. 2025 Aug 22;25:1059. doi: 10.1186/s12879-025-11299-2 (PMC12374319; doi:10.1186/s12879-025-11299-2)
Supplement: Supplementary file 1 — Supplementary Material 1. [file 12879_2025_11299_MOESM1_ESM.docx]

**Questionnaire**

1. Dose your child have any diseases as the following (multiple choice)?

□ Atopic dermatitis

□ Allergic conjunctivitis

□ Allergic rhinitis

□ Asthma (hyperactivity airway)

□ Immunodeficiency

□ Malignancy

□ Congenital abnormal facial development

□ Received head surgery before

□ Have bacterial paranasal sinusitis for more than 28 days

□ Others: _____________________

1. Dose your child blow the noses when having nasal symptoms?

□ Never

□ Seldom (less than once per day in average)

□ Often (once per day)

□ Very frequent (more than twice per day)

1. Dose your child irrigate the noses when having nasal symptoms?

□ Never

□ Seldom (less than once per day in average)

□ Often (once per day)

□ Very frequent (more than twice per day)

1. What is the care way of your child in the daytime?

□ Parents

□ Babysitter

□ Kindergarten

□ School

□ Others: _______________

1. How many children(<19 years old) live at your home (including the index case): _______
2. How many adults (<19 years old) live at your home: _______
3. Do you have the habit of burning incense to worship your ancestors at home?

□ Every day

□ On special festivals

□ No

1. Is there secondhand/thirdhand smoke in your home?

□ Yes

□ No

1. Do you have pets at home (multiple choice)?

□ Dog

□ Cat

□ Others: _____________________

1. Has your child received antibiotics in the past three months?

□ Yes

□ No

□ Unsure

1. Did your child receive three doses of 13-valent pneumococcal conjugated vaccine?

□ Yes

□ No

□ Unsure

1. Does your child receive influenza vaccine every year?

□ Every year

□ Not every year

□ Never

□ Unsure

1. The body weight ______kg and body height _______cm of your child.
2. The education level of father:

□ Illiterate or elementary

□ Junior high school

□ Senior high school

□ College

□ Graduate school

1. The education level of mother:

□ Illiterate or elementary

□ Junior high school

□ Senior high school

□ College

□ Graduate school

1. The total monthly household income of your family:

□ Less than fifty thousand

□ Fifty thousand to one hundred thousand

□ One hundred thousand to two hundred thousand

□ Two hundred thousand to three hundred thousand

□ More than three hundred thousand

1. Your area of ​​residence is:

□ Taipei City □ New Taipei City □ Kaohsiung City □ Yilan County

□ Taoyuan City □ Hsinchu County □ Hsinchu City □ Miaoli County

□ Taichung City □ Changhua County □ Nantou County □ Yunlin County

□ Chiayi County □ Tainan City □ Pingtung County □Taitung County

□ Hualien County □ Penghu County □ Keelung City □Chiayi City

□Kimma Area

Thank you very much!!
